# ABL209 (NEOK002): Designing an EGFR × MUC1 Bispecific TOP1i ADC with Promising Antitumor Activity and Enhanced Therapeutic Window

**DOI:** 10.1158/1535-7163.MCT-26-0088

**Published:** 2026-06-15

**Authors:** Bora Lee, Hyeon Ji Park, Byeong Min Yoo, Hangil Kim, Arim Seo, Youngeun Hong, Yo-Seob Lee, Donghoon Yeom, Yong-Gyu Son, Jaehyun Eom, Daehae Song, Byungje Sung, Jinhyung Ahn, Junyoung Kim, Weon-Kyoo You, Sang Hoon Lee, Jinwon Jung

**Affiliations:** ABL Bio Inc., Seoul, Republic of Korea.

## Abstract

ABL209 (NEOK002) is a bispecific antibody–drug conjugate (ADC) designed to enhance efficacy and therapeutic window through dual targeting of the epidermal growth factor receptor (*EGFR*) and mucin 1 (*MUC1*). EGFR is a key oncogenic driver in multiple tumor types; however, clinical targeting of EGFR is limited by dose-dependent skin toxicity. MUC1 is a tumor-associated antigen characterized by aberrant glycosylation and overexpression, but its expression can be heterogeneous, and the MUC1 extracellular domain is shed from the tumor, limiting monospecific targeting of MUC1. ABL209 is a heterodimeric 1 + 1 bispecific ADC with a drug-to-antibody ratio of 4, conjugated with tavatecan. ABL209 demonstrated enhanced cell binding and internalization compared with monospecific EGFR or MUC1 ADCs. ABL209 did not seem to inhibit the proliferation of human epidermal keratinocytes *in vitro*, unlike cetuximab-based ADCs. *In vivo*, ABL209 resulted in complete regression of tumors with single doses as low as 1.5 mg/kg in a CFPAC-1 pancreatic cell line–derived xenograft model. ABL209 demonstrated tumor growth inhibition across all 36 tested patient-derived xenograft models, inducing tumor regressions in 78% of models and showing efficacy in 6 of 10 *KRAS*-mutant tumors. Cotreatment with sotorasib prolonged tumor regression in a *KRAS*-mutated NCI-H1373 model for 58 days following treatment. ABL209 showed a favorable pharmacokinetic profile with a half-life of 5.2 days at 10 mg/kg in monkeys. ABL209 was well tolerated in monkeys up to 40 mg/kg. Our data suggest that a bispecific ADC leverages the benefits of cotargeting two antigens, resulting in enhanced antitumor activity while reducing liabilities through attenuated target-related toxicities.

## Graphical Abstract

**Figure:**
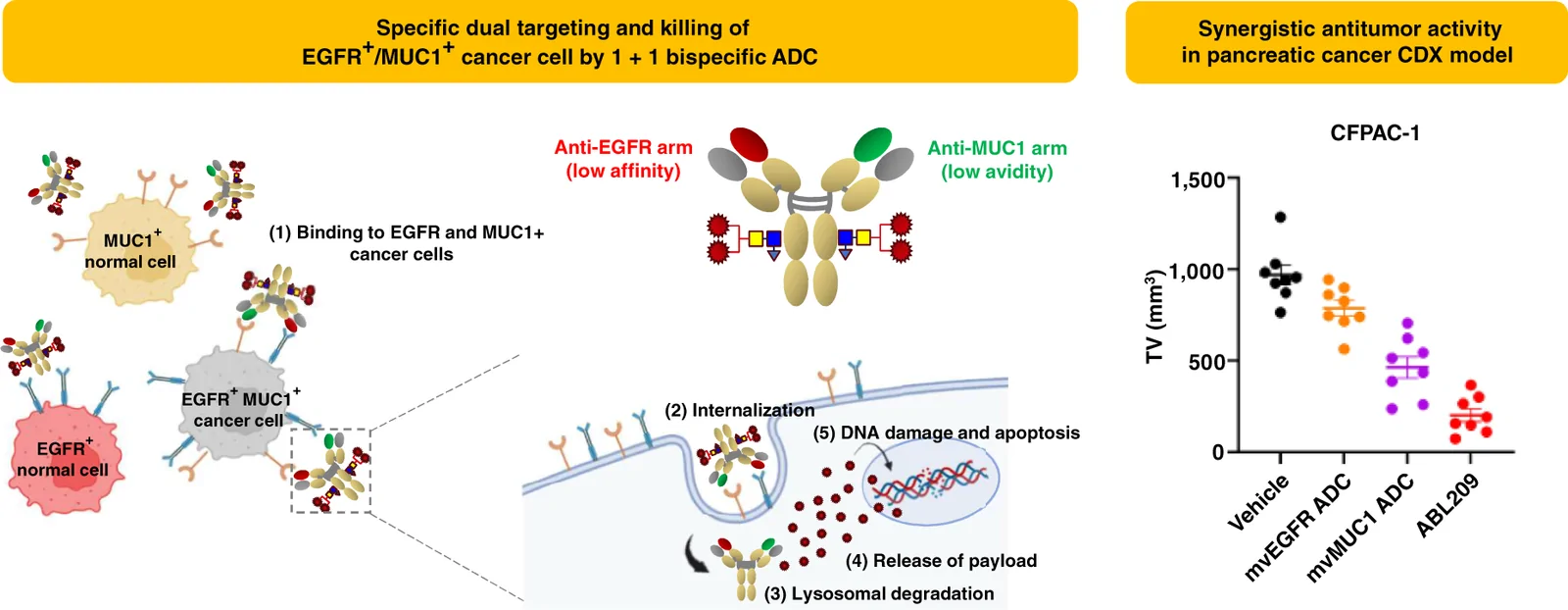

## Introduction

The epidermal growth factor receptor (*EGFR*) is a 1,186–amino acid transmembrane receptor tyrosine kinase that plays a critical role in regulating cell growth, proliferation, and survival not only in normal tissue but also in cancer. Modulating EGFR has been an established therapeutic strategy for anticancer drug development (1). EGFR tyrosine kinase inhibitors (TKI), including osimertinib, have revolutionized non–small cell lung cancer (NSCLC) treatment. Anti-EGFR antibodies, including cetuximab and panitumumab, bind to EGFR and inhibit EGF-mediated dimerization and signaling, thereby suppressing cancer growth. These antibodies are clinically vital options for advanced colorectal and head and neck cancers.

Although these innovative medicines have improved patient treatment, this approach still encounters several limitations. Cancers can bypass EGFR inhibition by anti-EGFR antibodies if tumors possess downstream cancer-driving mutations in *KRAS* or *BRAF*. TKIs are only applicable to patients with matching actionable genetic alterations (AGA), including exon 19 deletion and/or T790M mutation. Moreover, such AGAs are limited to NSCLC and are not observed in other types of indications. Even in NSCLC, a substantial portion of patients have wild-type EGFR and do not present AGA (2). Therefore, a novel therapeutic approach is required to overcome resistance to conventional therapies and improve clinical benefits in NSCLC and other indications.

Inhibiting EGFR can also induce on-target toxicity because EGFR signaling is important for the homeostasis of normal tissue, including skin keratinocytes and epithelial cells (3, 4). The anti-EGFR antibodies are associated with an 80% incidence of skin rash (5) and a 70% incidence of gastrointestinal tract–related toxicity (6). Most of these are low-grade and not life-threatening, but they reduce the quality of life of the patient and can lead to discontinuation or dose modification (7). Therefore, a new modality that can target and eliminate EGFR-expressing cancer cells while sparing normal cells, thereby avoiding EGFR inhibition–related toxicity, might help fulfill the clinical unmet need for EGFR-related tumors.

Mucin 1 (*MUC1*) is a 1,255-amino acid–long mucin protein that is highly glycosylated and forms a protective mucinous layer on the apical membrane of highly polarized epithelial cells. MUC1 is often overexpressed in multiple cancers (8), and its atypical expression leads to hypoglycosylation and the generation of neoantigens (tumor-associated MUC1 and TA-MUC1; refs. 9, 10). MUC1 is a single-pass transmembrane protein, but it can be cleaved by ADAM metallopeptidase domain 17 (11). There are also two secreted isoforms of MUC1 produced by alternative splicing in the tumoral context. The presence of neoantigens makes MUC1 an ideal target for cancer therapy (12, 13), and the circulating form of these neoantigens makes MUC1 an ideal cancer biomarker (10, 11). However, the presence of the soluble form represents a significant challenge in the development of MUC1-targeting therapeutic agents, as a shed soluble MUC1 protein in the circulation may sequester or neutralize the targeting agent (13). This possible sink effect may prevent MUC1 antibodies from reaching the actual tumor-bound MUC1 on cancer cells and thus may reduce treatment effectiveness. Therefore, new epitope strategies might need to be considered for targeting MUC1.

In this study, we describe a new bispecific antibody–drug conjugate (ADC) that addresses both problems. The new ADC, ABL209 (also known as NEOK002), is a bispecific ADC targeting both EGFR and MUC1, with a distinctive EGFR-binding property (less affinity than cetuximab) and a MUC1 epitope. There are several reports that MUC1 is linked with EGFR in cancer biology (14–18). Therefore, tumor specificity may be enhanced by combining these two targets in ADC development. We demonstrate that these characteristics make ABL209 more specific against dual tumor antigens while reducing potential on-target toxicity.

## Materials and Methods

### Cell lines

All cancer cell lines were purchased from vendors in the period from 2023 to 2025. All cell lines were cultured in the recommended media under standard CO_2_ incubator conditions and routinely authenticated by morphology and growth characteristics and were confirmed to be *Mycoplasma*-free. Several cell lines were not tested for *Mycoplasma*, and this is acknowledged as a limitation of the study. All cell lines were authenticated by the suppliers using short tandem repeat profiling, and master cell banks and working cell banks were prepared immediately and stored in liquid nitrogen. The working cell banks were used within 18 passages after thawing. The detailed information of each cell line is described in Supplementary Table S1.

### Antibodies, ADCs, and reagents

The antibodies were obtained by immunization of RenLite mice, which are transgenic mice with human immunoglobulin genes. The immunogens were EGFR extracellular domain (ECD; 25-645) or MUC1 ECD (24-1158). Single B-cell screening was done using the Beacon optofluidic system (Bruker Cellular Analysis). The recombinant antibodies were expressed in HEK293 or CHO cells and purified with affinity chromatography (MabSelect SuRe, Cytiva) and size exclusion chromatography (Superdex200, Cytiva). ADCs were conjugated with SYNtecan E using Synaffix’s GlycoConnect technology (19), and the resulting ADCs were analyzed using size exclusion-ultra performance liquid chromatography and reverse phase-ultra performance liquid chromatography to determine their purity and drug-to-antibody ratio (DAR; Supplementary Figs. S1 and S2). An isotype control ADC with the same linker payload was produced and showed a comparable DAR. Sotorasib (HY-114277), osimertinib (HY-15772), and datopotamab deruxtecan (HY-141598) were purchased from MedChemExpress. The antibody sequence of naked ABL209 is shown in Supplementary Fig. S3.

### ELISA

The recombinant MUC1 antigens, including MUC1 (890-1158; Sino Biological), MUC1 (1036–1155; ACROBiosystems), and MUC1 (1098–1158; ACROBiosystems), were coated onto 96-well plates at 1 µg/mL in PBS and incubated overnight at 4°C. After blocking with 1% BSA in PBS, antibody samples were applied in a 10-point, 4-fold serial dilution starting at 100 nmol/L and incubated for 2 hours at 37°C. Bound antibodies were detected using horseradish peroxidase (HRP)–conjugated anti-human IgG F(ab′)_2_ secondary antibody (RRID: AB_228273), followed by 3,3′,5,5′-Tetramethylbenzidine substrate (Sigma). The reaction was stopped with 0.5 N sulfuric acid (Samchun), and the absorbance was measured at 450 nm. The EC_50_ values were calculated using a four-parameter logistic (4PL) model in GraphPad Prism (version 10.6.1).

### Surface plasmon resonance

The binding kinetics of antibodies to their target antigens were determined using surface plasmon resonance (SPR) on a Biacore T200 instrument (Cytiva). The antibodies were used as ligands, and the target antigens were analytes. The antibodies were captured onto the chip surface via immobilized Protein A (Series S Sensor Chip Protein A, Cytiva). The target capture levels were set to approximately 30 RU for measuring affinity against the human and rhesus monkey EGFR and approximately 200 RU for measuring affinity against the human and cynomolgus monkey MUC1.

All binding affinity analyses were conducted at 25°C in HBS-EP buffer [1×, 10 mmol/L of HEPES, 150 mmol/L of NaCl, 3 mmol/L of EDTA, 0.05% surfactant P20 (pH 7.4; Cytiva, BR100669)]. Recombinant antigens [human EGFR (ACROBiosystems, EGR-H5222; 25–645)], rhesus monkey EGFR (ACROBiosystems, EGR-C52H1; 25–645), human MUC1 (ACROBiosystems, MU1-H52H5; 1036–1155), and cynomolgus monkey MUC1 (ACROBiosystems, MU1-C52H5; 254–373)] were the analytes and were injected in a 2-fold serial dilution ranging from 12.5 to 200 nmol/L (including a 0 nmol/L blank) using a multicycle kinetics format. The analytes were injected at a flow rate of 30 μL/min, with an association phase of 60 seconds and a dissociation phase of 180 seconds.

Following each analyte injection cycle, the sensor surface was regenerated by a 30-second pulse of Glycine 1.5 (Cytiva, BR100354) at the same flow rate, followed by equilibration with HBS-EP buffer. The sensorgrams were double-referenced by subtracting the reference flow cell response and blank injection.

The kinetic parameters, including the association rate constant (*k*_*a*_), the dissociation rate constant (*k*_*d*_), and the equilibrium dissociation constant (K_D_), were determined by globally fitting the processed sensorgram data to a 1:1 Langmuir binding model using Biacore T200 Evaluation Software (version 3.2.1).

### Flow cytometry

The cells were harvested, washed, and seeded in a 96-well plate. The test articles were serially diluted in assay buffer. The samples were incubated with cells for 1 hour at 4°C and washed twice. The cells were then stained with anti-human IgG (Fc-specific)–FITC antibody (Sigma-Aldrich, RRID: AB_259808) or mouse anti-human IgG (Fab-specific)–PE antibody (Invitrogen, RRID: AB_2536766) for 1 hour at 4°C. After two final washes, the fluorescence was analyzed via flow cytometry (BD LSRFortessa system, BD Biosciences).

### Cytotoxicity assay

The cells were seeded in 96-well plates at densities specified for each cell line and incubated for approximately 6 hours under their respective culture conditions before treatment. The test articles were serially diluted in complete culture medium and added to the wells in duplicate (final concentrations ranging from 250 nmol/L to 0.25 pmol/L). After 6 days of incubation, cell viability was assessed using the Cell Counting Kit-8 (CCK-8) assay (DOJINDO Laboratory), and absorbance was measured at 450 nm with background correction at 650 nm. The percentage of viable cells relative to untreated controls was calculated, and the IC_50_ values were determined via nonlinear regression analysis using a 4PL model in GraphPad Prism 10.

### Internalization assay

The cells were seeded in 96-well plates at densities specific to each cell line and incubated overnight at 37°C in a humidified atmosphere with 5% CO_2_. The test articles were mixed with Incucyte Fabfluor-pH Antibody Labeling Reagent (Sartorius, 8 µg/mL) and incubated at 37°C for 15 minutes, protected from light, before being added to the cells. Real-time internalization was monitored using the Incucyte Live-Cell Analysis System (Sartorius), with images acquired hourly using red fluorescence and phase-contrast channels (10× objective). Quantitative image analysis was performed using Incucyte software.

### Proliferation assay

The HT-29 cells were maintained in McCoy’s 5A medium supplemented with 10% FBS and antibiotic–antimycotic (Gibco) at 37°C with 5% CO_2_. For proliferation assays, cells were seeded into 96-well plates and treated with serially diluted antibodies prepared in serum-free McCoy’s 5A containing 200 nmol/L of human EGF (Cell Signaling Technology). After 72 hours, cell viability was measured using the CCK-8 (DOJINDO) according to the manufacturer’s instructions. The absorbance at 450 nm was quantified, and data were analyzed using GraphPad Prism.

### Western blotting

The A-431 cells were serum-starved and pretreated with the test articles prior to stimulation with recombinant human EGF. A standard Western blotting protocol was followed. Primary antibodies against phospho-EGFR (Cell Signaling Technology, RRID: AB_2096270), total EGFR (Cell Signaling Technology, RRID: AB_2246311), and GAPDH (Cell Signaling Technology, RRID: AB_1642205) were used. An HRP-conjugated anti-rabbit IgG secondary antibody (Cell Signaling Technology, RRID: AB_2099233) was used for detection. Protein bands were visualized by electrochemiluminescence and imaged using a ChemiDoc system (Bio-Rad Lab. Inc.).

### Confocal microscopy

The cells were incubated overnight and treated with 50 nmol/L of antibody for 1 hour at 4°C for binding. Internalization was then induced for 24 hours at 37°C in protease inhibitor–containing media. The cells were fixed with 4% PFA, then permeabilized with 0.1% Triton X-100 for 15 minutes at room temperature and blocked with 5% NGS. Internalization was analyzed via confocal microscopy after staining with anti–lysosomal-associated membrane protein 1 (LAMP1) primary antibody, followed by Alexa Fluor 488 anti-human IgG (Thermo Fisher Scientific, RRID: AB_2534080) and Alexa Fluor 568 antimouse IgG (Thermo Fisher Scientific, RRID: AB_2535804) secondary antibodies. The former was used to detect the target ADC and the latter for LAMP1.

### Western blot analysis of DNA damage and apoptosis markers

The MDA-MB-468 cells were seeded in a six-well culture plate and incubated overnight at 37°C. The cells were treated with test articles (final concentration 100 nmol/L) or exatecan mesylate (MedChemExpress, 10 nmol/L) for the indicated time. After incubation, the cells were harvested, washed with PBS, and lysed in RIPA buffer supplemented with a protease inhibitor (Thermo Fisher Scientific) and a phosphatase inhibitor (Thermo Fisher Scientific), 5 mmol/L of EDTA, and 1% SDS (Welgene). The lysates were clarified by centrifugation, and protein concentrations were determined via the BCA assay. Briefly, 10 µg of protein was subjected to SDS-PAGE and transferred to polyvinylidene difluoride membranes (Invitrogen). The membranes were blocked with 4% BSA in TBS with Tween 20 and probed with antibodies against phosphohistone H2AX (Cell Signaling Technology, RRID: AB_2118009), histone H2AX (Cell Signaling Technology, RRID: AB_10860771), phospho-Chk1 (Cell Signaling Technology, RRID: AB_331212), Chk1 (Santa Cruz Biotechnology, RRID: AB_627257), cleaved PARP (*PARP1*; Cell Signaling Technology, RRID: AB_10699459), cleaved caspase-3 (*CASP3*; Abcam, RRID: AB_725947), and rabbit anti–β-actin mAb (Cell Signaling Technology, RRID: AB_1903890), followed by anti-rabbit IgG HRP-conjugated secondary antibody (Cell Signaling Technology, RRID:AB_2099233) and anti-mouse IgG HRP-conjugated secondary antibody (Cell Signaling Technology, RRID: AB_330924). Signals were detected using an enhanced chemiluminescence substrate (Thermo Fisher Scientific) and visualized using a ChemiDoc MP Imaging System (Bio-Rad). For total protein detection, membranes were stripped and reprobed with the corresponding antibodies.

### Antibody-dependent cellular cytotoxicity reporter bioassay

The MDA-MB-468 cells were seeded in a 96-well culture plate and incubated overnight at 37°C. The cells were then coincubated with effector cells (Promega) and serially diluted test articles in antibody-dependent cellular cytotoxicity assay buffer [RPMI 1640 (ATCC) supplemented with 0.5% ultralow IgG FBS (Gibco)] for 24 hours at 37°C. Following incubation, Bio-Glo Luciferase Assay Reagent (Promega) was added, and luminescence was measured using a PHERAstar FS plate reader (BMG LABTECH).

### Complement-dependent cytotoxicity bioassay

The MDA-MB-468 cells were seeded in a 96-well culture plate and incubated overnight at 37°C. The cells were then coincubated with serially diluted test articles in complement-dependent cytotoxicity assay buffer [L-15 (ATCC) supplemented with 4% ultralow IgG FBS (Gibco)] and human complement serum (Quidel) for 5 hours at 37°C. To assess cell death, CytoTox-Glo Reagent (Promega) was added, and luminescence was measured using a PHERAstar FS plate reader (BMG LABTECH).

### Cell-cycle analysis

The MDA-MB-468 cells were seeded in a six-well culture plate and incubated overnight at 37°C. The cells were treated with test articles (final concentration 1 nmol/L) for 48 hours. Both floating and adherent cells were collected, washed with PBS, and fixed in 70% ethanol at 4°C for at least 2 hours. Prior to staining, the cells were washed with PBS and resuspended in propidium iodide (PI) and RNase A staining solution. After incubation at 37°C for 30 minutes in the dark, the DNA content was analyzed via flow cytometry, and data were processed using FlowJo software.

### Annexin V/PI apoptosis assay

The MDA-MB-468 cells were seeded in a six-well culture plate and incubated overnight at 37°C. The cells were treated with test articles for 3 days. Both floating and adherent cells were collected, washed twice with cold PBS, and resuspended in 1× binding buffer. The aliquots (5 × 10^5^ cells) were stained with APC Annexin V and PI (BioLegend) according to the manufacturer’s protocol. After incubation for 15 minutes at room temperature in the dark, 1× binding buffer was added, and samples were analyzed via flow cytometry within 1 hour. The data were processed using FlowJo software. Early and late apoptotic populations were determined based on Annexin V/PI staining profiles.

### HMGB1 immunoassay

The MDA-MB-468 cells were seeded in a 96-well culture plate and incubated overnight at 37°C. The cells were treated with test articles (final concentration 100 nmol/L) for 48 hours. The HMGB1 levels in the culture supernatant were measured using the Lumit HMGB1 Immunoassay Kit (Promega) according to the manufacturer’s protocol. Briefly, after incubation with the anti-hHMGB1 Antibody Mixture and Lumit Detection Reagent B, luminescence was measured using a PHERAstar FS (BMG LABTECH). Cell viability was assessed in parallel using the CellTiter-Glo Luminescent Cell Viability Assay (Promega). Relative luminescence units were recorded, and data were analyzed using GraphPad Prism 10 software.

### Calreticulin exposure analysis

The MDA-MB-468 cells were treated with test articles for 48 hours. After treatment, the cells were collected and stained with Zombie Violet Fixable Viability Dye (BioLegend, 423113, 1:500). The cells were then incubated with an anti-calreticulin (CALR) antibody (Invitrogen, PA3-900, RRID: AB_325990, 1:100 in FACS buffer), followed by fixation with BD Cytofix (BD Biosciences, 554655). Fixed cells were stained with an Alexa Fluor 546–conjugated goat anti-rabbit secondary antibody (Invitrogen, A-11010, RRID: AB_2534077, 1:250). The samples were analyzed via flow cytometry, and viable (Zombie Violet–negative) CALR-positive cells were quantified using FlowJo software.

### *In vitro* bystander killing effect assay

The bystander effect was assessed using cocultures of EGFR-positive MDA-MB-468 and EGFR-negative MDA-MB-453 cells mixed at N/P ratios of 0:1, 1:0, or 1:1. After 6 hours of preincubation, ADCs or free drug (serial 4-fold dilutions from 10 nmol/L) were added, and cells were cultured for 6 days. The total cells were collected, stained with Fixable Viability Dye eFluor 780 (Invitrogen, 1:1,000) and PE anti-human EGFR antibody (BioLegend, RRID: AB_10896794, 1:500), and analyzed via flow cytometry to quantify antigen-positive and antigen-negative populations.

### Caspase-3/7 apoptosis assay

The MDA-MB-468 cells were seeded in a 96-well culture plate (Thermo Fisher Scientific) and incubated overnight at 37°C with 5% CO_2_. The cells were treated with test articles in the presence of Caspase-3/7 Green reagent (Sartorius, 1:2,000). Real-time apoptosis was monitored using the Incucyte Live-Cell Analysis System (Sartorius) with phase-contrast and green fluorescence channels. Images were acquired every hour with a 10× objective, and quantitative analysis was performed using Incucyte software.

### Single-cell RNA sequencing in PDX models

Single-cell RNA sequencing (scRNA-seq) libraries were constructed using the GEXSCOPE Single Cell RNA Library Kit. Raw FASTQ files were processed with Cell Ranger (version 6.1.2) using the GRCh38/mm10 reference to generate expression matrices, which were imported into Seurat for quality control and downstream analysis. The feature–barcode matrix was converted into a Seurat object using the Seurat package (version 4.0) for quality control and downstream analysis. Cells with 5,000 detected genes or >20% mitochondrial gene content were excluded. The data were normalized and scaled, and the top 2,000 variable genes were used for principal components (PC) analysis. Clustering was performed using the top 20 PCs (resolution 0.1), and Uniform Manifold Approximation and Projection for Dimension Reduction was used for visualization. The FindAllMarkers function was used to identify cell markers in each cluster for cell population annotation.

### Human tissue microarray

Tissue microarrays (TMA) were purchased from TissueArray.com LLC and analyzed using the Roche IHC/ISH Benchmark ULTRA system. The slides were stained with primary rabbit mAbs specific for EGFR (Abcam, ab227642) and anti-MUC1 antibody (Abcam, RRID: AB_2941824). The stained slides were scanned using an Aperio AT2 whole-slide scanner. A pathologist annotated tumor regions, excluding necrotic areas and artifacts. The H-scores were calculated by weighting the proportion of tumor cells at each staining intensity level using the following formula: (percentage of 3+ cells × 3) + (percentage of 2+ cells × 2) + (percentage of 1+ cells × 1). The immunohistochemistry (IHC) grades were determined according to the 10% rule. The samples were classified as 3+ when ≥10% of tumor cells exhibited strong (3+) staining. The samples were assigned a 2+ grade when ≥10% of cells showed at least moderate (2+) staining but fewer than 10% showed strong staining. The samples in which <10% of tumor cells demonstrated any positive staining (≥1+) were considered IHC 0 (negative).

### Mice

All the mouse studies using cell line–derived xenograft (CDX) models were approved by the Institutional Animal Care and Use Committee (IACUC) at ABL Bio Inc. in compliance with the guidelines established by the IACUC. The patient-derived xenograft (PDX) model screening studies using 36 subcutaneous HuPrime models were approved by the IACUC at Crown Bioscience, Inc. The studies using the T790M/C797S EGFR-mutated NSCLC model (CTG-4001) were approved by the IACUC at Champions Oncology. For all mouse tumor models described, 6- to 8-week-old female mice weighing 18 to 22 g were used. In all studies, mice were divided into treatment groups based on the tumor volume (TV) to achieve equal distribution between dosing groups. Given variation in tumor growth, all groups consisted of up to 10 mice to allow statistical comparisons between groups. The PDX screening study uses three mice per group. The tumor size and body weight of the mice were measured twice weekly. The body weight changes in mice across all efficacy studies are shown in Supplementary Fig. S4.

### CDX models

Human lung cancer cells (NCI-H1373 and NCI-H1975) or human pancreatic cancer cells (AsPC-1, HPAF-II, Capan-2, and CFPAC-1) were implanted (5 × 10^6^ cells/head) into the flanks of CB17 SCID mice (Janvier Labs). All cell lines used in the efficacy studies were confirmed to be *Mycoplasma*-free prior to implantation into animals. Each cancer cell suspension (PBS and Matrigel mixture, 200 μL/head) was inoculated subcutaneously into the right flank of the mice using a 1 mL syringe. When the average volume of tumors reached approximately 200 mm^3^, the mice were randomized into each treatment group (*N* = 3–8 mice/group) based on the TVs, and the test articles were administered once (day 1) via i.v. tail vein injection. The TV was calculated using the following formula: (length) × (width)^2^ × 0.5. When the average TV of a group reached 2,000 mm^3^, all mice in the group were sacrificed. Tumor growth inhibition (TGI) was determined by calculating the percent TGI: 100% × [1 − (final MTV − initial MTV of a treated group)/(final MTV − initial MTV of the control group)], where MTV indicates mean TV.

### PDX models

PDX screening across 36 subcutaneous HuPrime models was conducted using female BALB/c nude or NOD/SCID mice from GemPharmatech Co., Ltd. Mice were implanted subcutaneously into the right flank with patient-derived tumor fragments (2–3 mm in diameter) for tumor development. The randomization started when the MTV reached approximately 100 to 200 mm^3^ (*N* = 3 mice/group). Grouping was designated as day 0, and treatments were initiated on the same day as the study design. The body weight and TV data were measured as above.

The PDX study using the CTG-4001 human NSCLC xenograft model was conducted in female athymic nude immunocompromised mice from Charles River Laboratories. The fragments were implanted into the left flank of female athymic nude mice. The mice (*N* = 10 mice/group) were randomly assigned to treatment groups when tumors reached a mean volume of 214 mm^3^ (actual TV during randomization). ADCs were administered as a single intravenous dose, whereas osimertinib was given daily by oral gavage for up to 21 days. The body weight and TV data were measured as above. The TV and TGI were calculated as described in the previous section.

### Statistical analysis

The data for body weight (g) and TV (mm^3^) were calculated as mean ± SEM (standard error of mean) using Excel. The graphs for both parameters and all statistical analyses were generated using GraphPad Prism (GraphPad Software). The normality of the data was assessed using the Shapiro–Wilk test. For CDX models, multiple group comparisons with the control group were performed using the Kruskal–Wallis test followed by the Dunn multiple comparisons test. For the CTG-4001 model, multiple group comparisons with the control group were performed using the Kruskal–Wallis test followed by the Dunn multiple comparisons test. The *P* values <0.05 were considered statistically significant.

## Results

### Structural and functional characterization of ABL209

**Figure 1.**
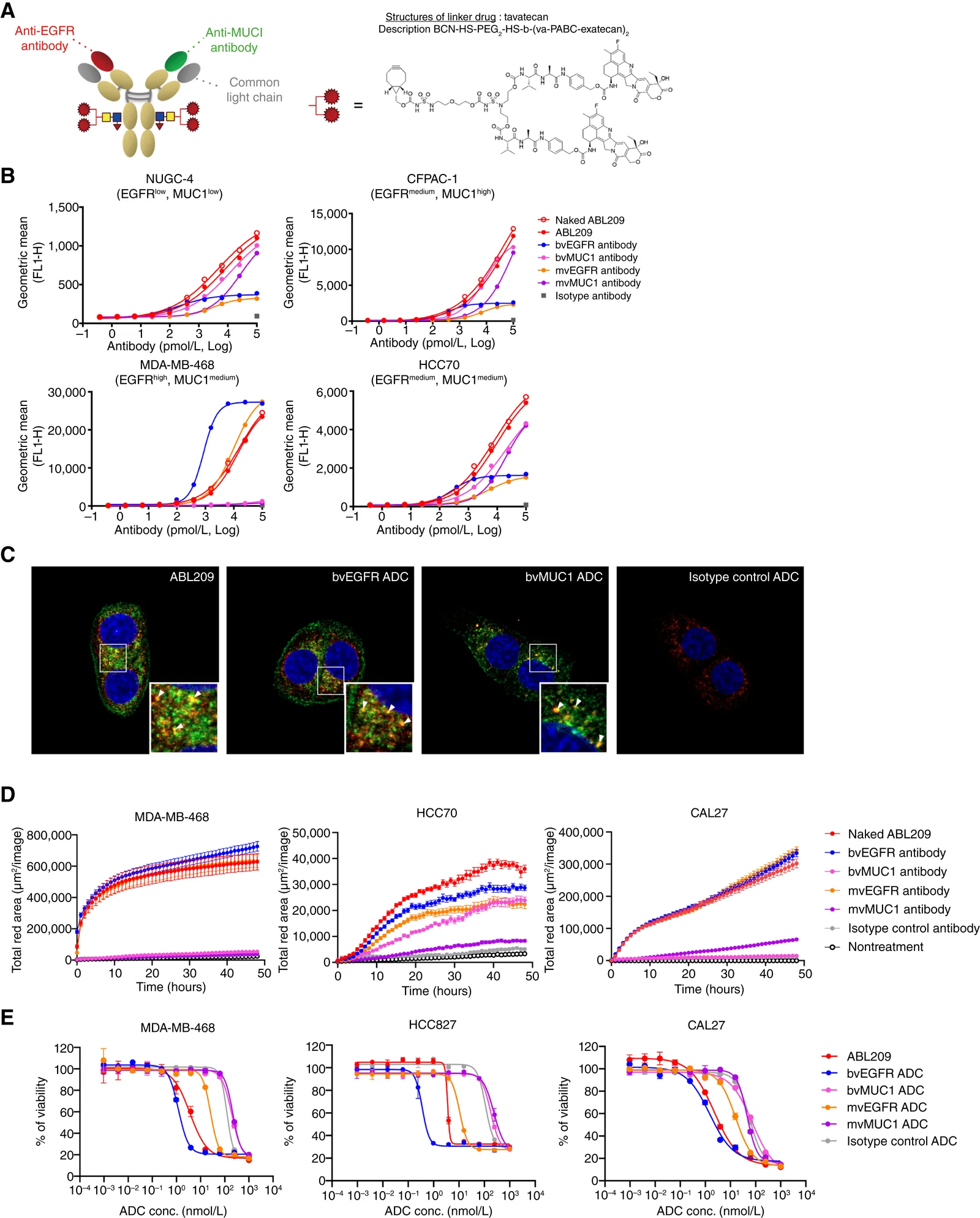
Characterization of ABL209. **A,** Schematic structure of ABL209. **B,** Cell surface binding of ABL209, naked ABL209, monospecific antibodies, and isotype control antibody in various cancer cell lines. **C,** Internalization analysis using confocal microscopy in HCC70 cells. DAPI staining of the cell nuclei was visualized in blue. ADC (green) can be detected on the cell surface and in the lysosomal trafficking marker-positive compartments (red). Colocalization to lysosomes is depicted by yellow and marked by white triangles. **D,** Internalization of pHrodo-labeled antibodies and isotype control antibody. **E, ***In vitro* cell growth inhibition by ABL209, monospecific ADCs, and isotype control ADC.

A schematic structure of ABL209 is shown in Fig. 1A. ABL209 is a 1 + 1 bispecific ADC targeting EGFR and MUC1. Its parental clones were derived from the immunization of humanized mice with a common light chain (RenLite mouse, Biocytogen; ref. 20). The immunogens were the full-length ECD of EGFR or MUC1. Hit clones were further screened by their affinity, tumor cell binding, internalization, and species cross-reactivity. The lead EGFR clone was selected because it has a weak binding affinity, but it showed a reasonable cytotoxic effect as an ADC. The clone also showed reduced internalization in a monovalent format. We expected that these properties could reduce EGFR-related toxicity. For MUC1, only one clone passed all the criteria above. The selected EGFR and MUC1 binders were combined in a heterodimeric IgG molecule with the common light chain using a knob-in-hole design (21). Therefore, it is designed to bind one arm to EGFR and the other to MUC1 as a bispecific binder while being monovalent for each target.

The ADC is site-specifically conjugated with a cleavable linker-payload, bicyclononyne carbamoyl sulfamide Val-Ala-PABC exatecan (SYNtecan E, INN: tavatecan), at asparagine 297 of the heavy chain via enzymatic glycan remodeling and metal-free click chemistry, using Synaffix’s GlycoConnect technology (19). The resulting EGFRxMUC1 ADC has a theoretical DAR of 4 and an average DAR of approximately 3.8, providing homogeneous conjugation, whereas modification of the N-glycosylation site eliminates Fcγ receptor binding, thereby reducing effector function (Supplementary Fig. S5).

The epitope of MUC1 is illustrated schematically in Supplementary Fig. S6. The ELISA result also showed that ABL209 bound only to constructs that have the Sea urchin sperm protein, Enterokinase, and Agrin domain and did not bind to the ectodomain of MUC1-C (Supplementary Fig. S7). In cell microarray screening, ABL209 bound to MUC1 isoform 1 (UniProt: P15941-1, full-length form) and isoform 8 (UniProt: P15941-8, variable number of tandem repeat truncated form with SEA domain) but not to isoform Y (UniProt: P15941-7), which is an N-terminus truncated form and does not have an SEA domain. Therefore, we defined the SEA domain as the epitope of ABL209. For the EGFR side, the cell microarray test showed that ABL209 also bound to EGFR vIII but did not bind to the truncated form (UniProt: P00533-2), which has only domains I and II. The parental EGFR antibody showed binding competition with cetuximab. Therefore, we concluded that the epitope of ABL209 on EGFR is domain III (Supplementary Fig. S8). However, despite targeting a similar epitope as cetuximab, ABL209 was designed to minimize interference with EGFR signaling and mitigate potential on-target toxicity and displays more than 60-fold lower binding affinity against human and monkey EGFR with K_D_ of 54 and 67 nmol/L, respectively (Supplementary Fig. S9 and Supplementary Table S2). In contrast, most EGFR-targeting therapeutic antibodies, including cetuximab, bind to EGFR with high affinity (around 1 nmol/L; ref. 22). Unlike the EGFR antibody, the MUC1 antibody exhibited higher affinity for the MUC1 antigen (∼5 nmol/L) and demonstrated similar affinity for both human and cynomolgus monkey antigens.

Cell binding and internalization activity of ABL209 and the corresponding ADCs were evaluated using a panel of cell lines expressing varying levels of EGFR and MUC1. ABL209 consistently outperformed monospecific monovalent antibodies in binding affinity and binding level across most tested cell lines (Fig. 1B). This enhanced performance seemed to be attributed to its bispecific 1 + 1 architecture. However, this trend was not observed in MDA-MB-468 cells, in which extreme EGFR overexpression allowed the bivalent EGFR antibody to bind more strongly. The binding curve for MUC1 did not reach a plateau, unlike the EGFR arm within the tested concentration range. As the specificity and affinity of the MUC1 arm have been tested in several ways, including cell microarray and SPR, it is not due to nonspecific binding, but we do not have a definitive explanation yet. Building on these binding results, internalization efficiency was assessed by confocal microscopy using HCC70 cells (Fig. 1C). Confocal microscopy revealed that ABL209 exhibited markedly greater colocalization with LAMP-1 than the monospecific bivalent EGFR and MUC1 ADCs, demonstrating more efficient internalization and lysosomal trafficking compared with the monospecific bivalent EGFR and MUC1 antibodies. The cellular internalization of ABL209 was assessed using a pH-sensitive dye (pHrodo) and monitored in real time with the Incucyte Live-Cell imaging system in MDA-MB-468, HCC70, and CAL27 cell lines (Fig. 1D). The naked ABL209 showed better internalization than other antibodies in the HCC70 cell line. In the MDA-MB-468 and CAL27 cell lines, the naked ABL209 showed comparable internalization with EGFR antibodies. The differences in the internalization of bivalent antibodies were observed depending on the level of antigen expression on the cell surface. In cell lines such as MDA-MB-468 and CAL27, in which EGFR is expressed at substantially higher levels than MUC1, the EGFR antibody showed markedly stronger cellular internalization than the MUC1 antibody. In contrast, in cell lines such as HCC70, in which EGFR and MUC1 are expressed at comparable levels, the difference in internalization between the EGFR and MUC1 antibodies was less pronounced than that observed in the former cell lines. Consistent with the confocal microscopy results, ABL209 efficiently internalized into target tumor cells, showing robust intracellular uptake comparable to that of monospecific bivalent ADCs.

The cytotoxic activity of ABL209 was further evaluated in comparison with monospecific ADCs in a series of *in vitro* assays using human cancer cell lines. ABL209 exhibited an IC_50_ in the range of 80.2 to 2.0 nmol/L, which is 2- to 37-fold stronger than the isotype control ADC, consistent with its strong binding and efficient internalization properties. Notably, ABL209 demonstrated more potent *in vitro* cytotoxic activity than the corresponding monospecific monovalent ADCs and showed comparable potency to the monospecific bivalent ADCs (Fig. 1E; Supplementary Table S3).

### Induction of DNA damage and apoptosis by ABL209

**Figure 2.**
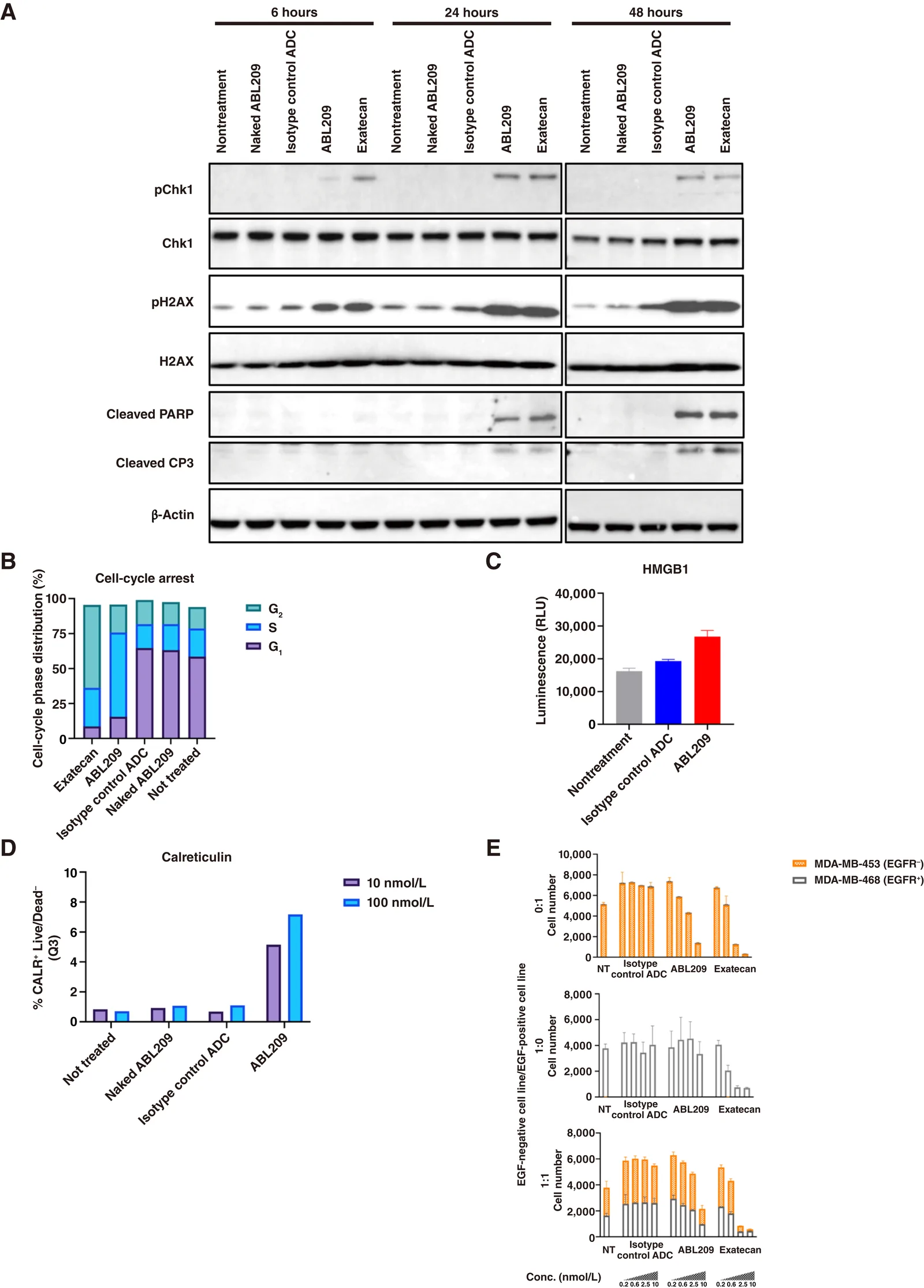
Induction of DNA damage and apoptosis by ABL209. **A,** Western blot analysis of DNA damage and apoptosis markers. MDA-MB-468 cells were treated with 100 nmol/L of naked antibody, ADCs, or exatecan. **B,** Cell-cycle analysis using MDA-MB-468 cells. Cells were treated with 1 nmol/L of naked antibody, ADCs, or exatecan. **C,** Representative histograms of HMGB1 release. MDA-MB-468 cells were treated with 100 nmol/L of ABL209 and isotype control ADC. **D,** CALR exposure in MDA-MB-468 cells. Cells were treated with 10 and 100 nmol/L of naked ABL209, isotype control ADC, and ABL209. **E,** Results of *in vitro* bystander effect. MDA-MB-468 (target-positive) and MDA-MB-453 (target-negative) cells were treated with exatecan or ADCs at 0.1, 0.6, 2.5, and 10 nmol/L. NT, not treated.

To assess the induction of DNA damage and apoptosis in target-expressing cell lines by ABL209, MDA-MB-468 cells were treated with ABL209, and the lysates were analyzed via Western blot for DNA damage markers (phosphorylated H2AX and Chk1) and apoptosis markers (cleaved PARP and caspase-3). Treatment with ABL209 or exatecan markedly increased the phosphorylation of H2AX and Chk1, as well as the cleavage of PARP and caspase-3 (Fig. 2A). We next evaluated the effects of ABL209 on cell-cycle progression in MDA-MB-468 cells. Treatment with ABL209 led to a marked accumulation of cells in the S- and G_2_-phase, consistent with its mechanism of action (Fig. 2B). Apoptosis induced by ABL209 was verified in a caspase-3/7 apoptosis assay and Annexin V/PI staining analysis (Supplementary Figs. S10 and S11).

### Induction of immunogenic cell death and bystander effects by ABL209

Immunogenic cell death (ICD) and bystander effects are considered key mechanisms that induce stronger and more durable responses when combined with immunotherapy agents. To assess the hallmark signals of ICD *in vitro*, HMGB1 release and CALR exposure were evaluated. HMGB1 release from MDA-MB-468 cells treated with ABL209 was measured using a luciferase-based bioluminescent assay. A 1.7-fold increase in HMGB1 release was observed following ABL209 treatment (Fig. 2C). The exposure of CALR on the cell surface was also assessed via flow cytometry, and treatment with ABL209 increased the CALR levels from 0.78% to 5.15% (10 nmol/L) or 7.18% (100 nmol/L; Fig. 2D). The bystander killing effect of ABL209 was assessed with a mixed culture assay and flow cytometry. MDA-MB-468 (EGFR^+^) and MDA-MB-453 (EGFR^−^) were tested following a 6-day treatment with the ADCs and exatecan under mono- and coculture conditions. As shown in Fig. 2E, antigen-negative MDA-MB-453 did not respond to ABL209 but did respond to exatecan (middle), whereas antigen-positive MDA-MB-468 did respond to ABL209 and exatecan. Under the coculture conditions, the numbers of viable MDA-MB-453 decreased in a dose-dependent manner with ABL209. These results indicate that ABL209 can kill both antigen-expressing and neighboring antigen-negative cells through payload diffusion or bystander effect, supporting its potential therapeutic benefit in tumors with heterogeneous antigen expression.

### Lack of interference with EGF-mediated cell proliferation and keratinocyte viability

**Figure 3.**
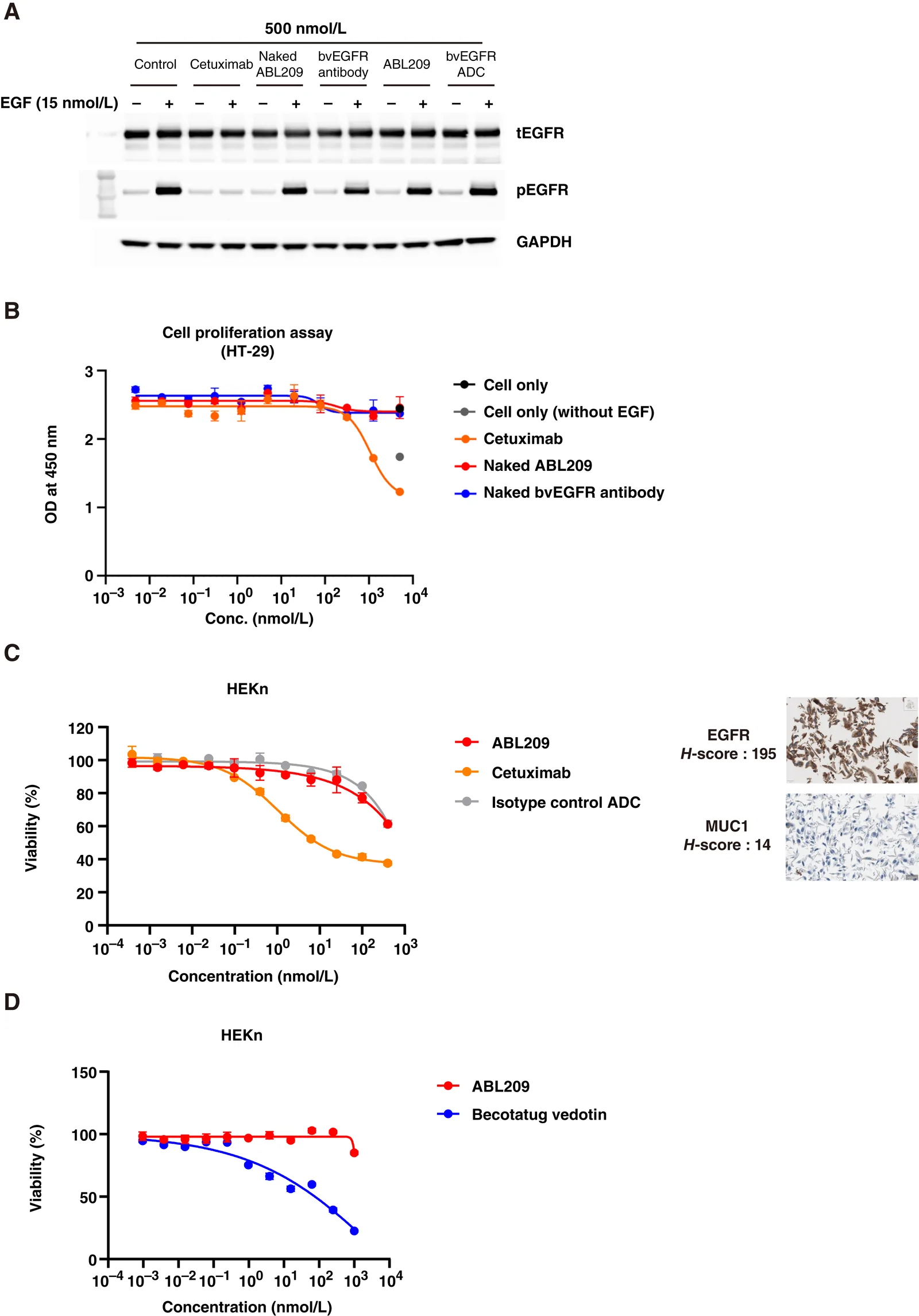
Optimized EGFR affinity and lack of EGFR signaling engagement. **A,** Western blot analysis of the effect on EGFR signaling. **B,** EGF-mediated proliferation of HT-29 using a CCK-8 assay. Cells were treated with cetuximab, naked bivalent (bv) EGFR antibody, or naked ABL209 and incubated at 37°C for 72 hours, after which cell viability was measured using CCK-8. **C,** The minimal effects on the proliferation of normal human keratinocytes. Cells were treated with cetuximab, a nonbinding control ADC, or ABL209 and incubated for 72 hours. **D,** The same experiment as in panel C was performed, with cells treated with becotatug vedotin or ABL209 and cultured for 6 days.

The inhibition of the EGFR signaling pathway induces EGFR-related on-target toxicity (23, 24). The inhibitory effect of ABL209 on EGFR signaling was assessed via Western blotting following EGF ligand stimulation (Fig. 3A). The stimulation with EGF increased the level of phosphorylated EGFR, and cetuximab treatment suppressed the EGFR phosphorylation almost completely. However, treatment with naked ABL209, its parental bivalent EGFR antibody, and their corresponding ADCs resulted in no discernible suppression of EGFR phosphorylation. This indicates that ABL209 had a negligible effect on the EGFR signaling pathway. The effect on the EGFR signaling pathway was further assessed by EGF-induced cell proliferation. The conjugated and unconjugated ABL209 did not inhibit the growth of EGF-treated HT-29 cells, whereas cetuximab inhibited cell proliferation at 1 µmol/L and above concentrations (Fig. 3B). Such observations were further extended to human primary keratinocytes in which growth inhibition leads to EGFR on-target toxicity. A cell proliferation assay was performed using human epidermal keratinocyte (HEKn) cells (neonatal; Fig. 3C). ABL209 and isotype control ADC showed minimal inhibition, maintaining more than 80% to 90% cell growth across all tested concentrations. In contrast, cetuximab markedly reduced cell growth in a dose-dependent manner down to 40%. The same experiment was repeated with an anti-EGFR ADC, becotatug vedotin, which uses a humanized cetuximab an antibody backbone. Becotatug vedotin also inhibited the proliferation of HEKn cells, and it was comparable to the effect observed with cetuximab (Fig. 3D). These results indicate that ABL209 did not interfere with the cell proliferation or survival of normal human keratinocytes, suggesting a lower risk of EGFR-related skin toxicity caused by high-affinity EGFR-inhibiting binders. To exclude the possibility that the lack of antiproliferative activity was due to insufficient EGFR binding by ABL209’s monovalent EGFR arm, we compared its binding affinity to human EGFR with that of a monospecific bivalent EGFR antibody by ELISA (Supplementary Fig. S12). Both antibodies exhibited concentration-dependent binding, and the bivalent antibody demonstrated stronger binding, as reflected by a lower EC_50_ value (0.045 nmol/L) compared with monovalent ABL209 (1.2 nmol/L). These findings indicate that although both antibodies effectively bind to EGFR, their distinct binding modes do not interfere with EGF-mediated proliferative signaling, thereby suggesting less skin-related side effects due to EGFR inhibition compared with cetuximab.

### Superior *in vivo* antitumor activity of 1 + 1 bispecific ADC compared with monospecific ADCs

**Figure 4.**
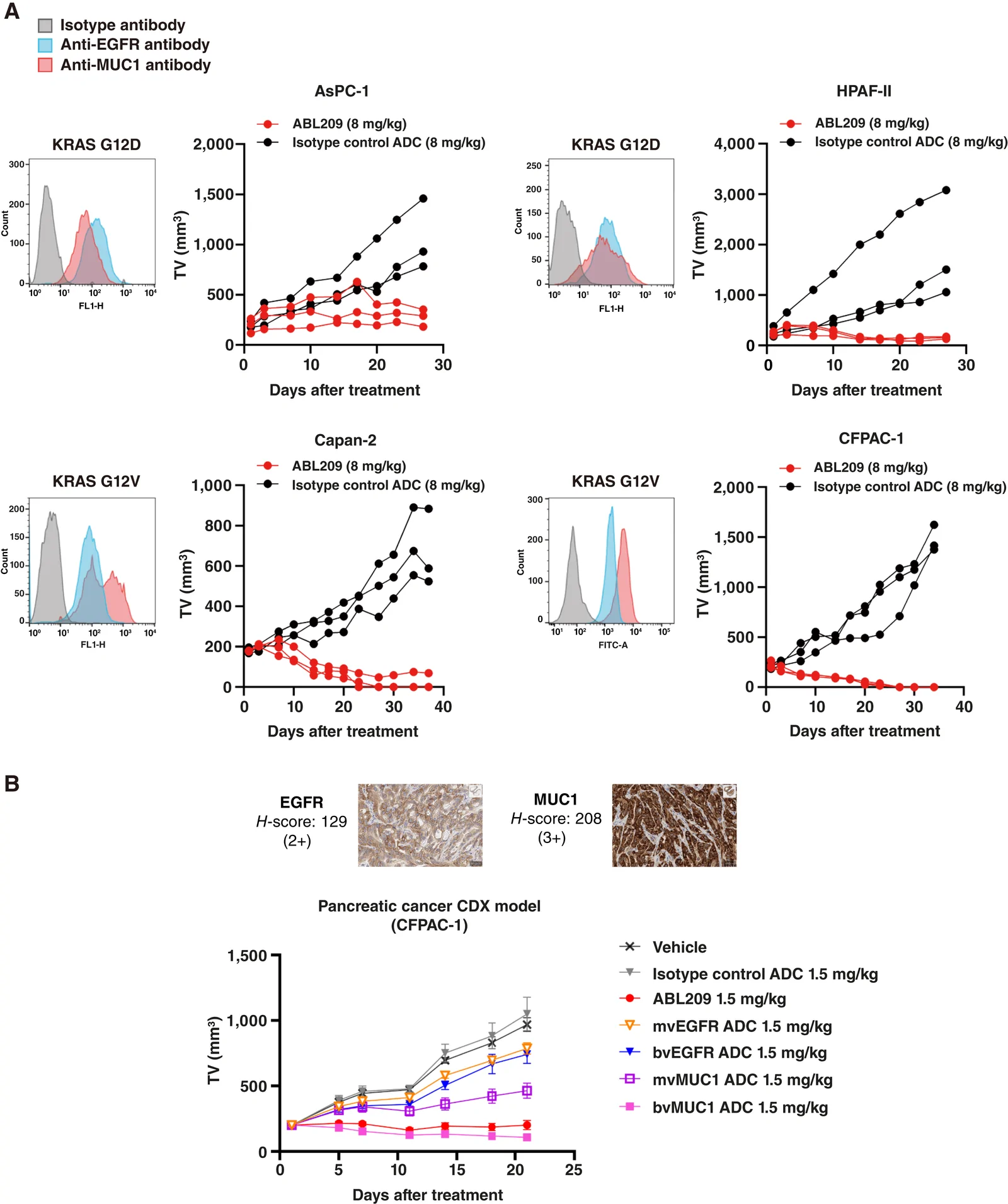
Evaluation of ABL209 efficacy and assessment of synergistic effects in pancreatic cancer cell lines. **A,** Preliminary efficacy study using *KRAS*-mutated pancreatic cancer cell lines. Mice inoculated with human cancer cells, AsPC-1, HPAF-II, Capan-2, or CFPAC-1 were intravenously administered 8 mg/kg isotype control ADC or ABL209 on day 1 (*N* = 3 mice/group). **B,** Synergistic antitumor activity of ABL209 in the CFPAC-1 CDX model (*N* = 8 mice/group). Mice were treated with ABL209, monovalent (mv) EGFR ADC with an inert arm, bivalent (bv) EGFR ADC, mvMUC1 ADC, bvMUC1 ADC, or isotype ADC. The dose was 1.5 mg/kg and a single dose.

To evaluate the efficacy of ABL209 in pancreatic cancer models, *in vivo* efficacy studies were conducted using four xenograft models that exhibit various EGFR and MUC1 expression and harbor *KRAS* mutations. ABL209 treatment resulted in marked tumor growth suppression compared with the isotype control across all four models including AsPC-1, HPAF-II, Capan-2, and CFPAC-1, with individual TVs for each mouse presented in Fig. 4A. These results demonstrate robust antitumor activity irrespective of antigen expression heterogeneity or oncogenic *KRAS* status.

To further assess the synergistic potency of ABL209 compared with monospecific ADCs, a comparative *in vivo* study was performed in the CFPAC-1 model. Prior to this study, a dose range–finding study was conducted using ABL209 to determine the minimum efficacious dose (MED) or suboptimal MED in which the difference in efficacy can be compared well, and 1.5 mg/kg was selected as the appropriate dose for subsequent efficacy evaluations. The 1 + 1 bispecific ADC demonstrated further enhanced efficacy relative to the monospecific monovalent EGFR ADC or MUC1 ADC, highlighting the increased therapeutic benefit conferred by bispecific engagement rather than the differences in format (Fig. 4B). To further substantiate the synergistic effect, an efficacy study of the same design was conducted in the NCI-H1373 cell line, and the results were consistent with the previous findings (Supplementary Fig. S13). Collectively, these results support the enhanced and synergistic therapeutic potential of the 1 + 1 bispecific format of ABL209 over monospecific monovalent ADC formats.

### Expanded PDX efficacy and target coexpression analysis

**Figure 5.**
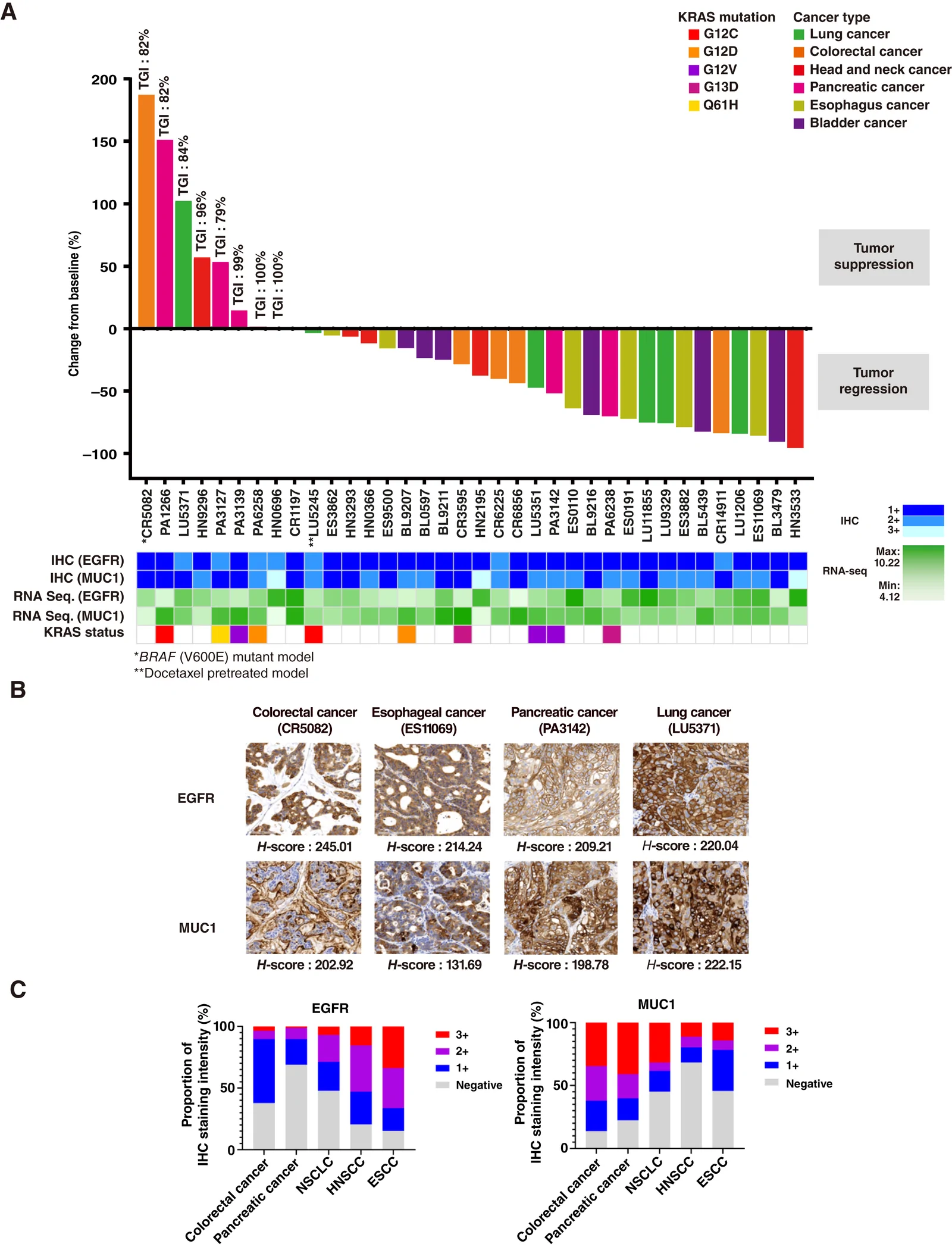
Antitumor activity of ABL209 in PDX models. **A,** Waterfall plot illustrating tumor responses to 10 mg/kg ABL209 treatment across 36 models (*N* = 3 mice/group). The respective cancer types are indicated by specific colors. TGI values are provided for models exhibiting tumor suppression, whereas models with negative values represent tumor regression. Protein expression levels for EGFR and MUC1 were evaluated by IHC and categorized into scores of 1+, 2+, and 3+. Green indicates gene expression levels (lower panel) representing log_2_-normalized RNA sequencing data. *KRAS* mutation variants are indicated by specific color coding at the bottom. **B,** Representative EGFR and MUC1 IHC staining data from various PDX models. **C,** IHC analysis results of the human tumor microarray. A total number of analyzed cores included 29 from colorectal cancer, 73 from NSCLC, 117 from head and neck squamous cell carcinoma (HNSCC), and 92 from esophageal squamous cell carcinoma (ESCC). Measurement of staining intensity of all annotated cells (0, 1+, 2+, and 3+) via image analyzer. IHC grades were assigned using the 10% rule: tumors with ≥10% of cells showing 3+ staining were graded as 3+; those with ≥10% showing ≥2+ but <10% showing 3+ were graded as 2+; and tumors with <10% of cells showing ≥1+ staining were graded as 0. Negative means not stained.

We conducted an expanded *in vivo* efficacy assessment of ABL209 in 36 PDX models representing six indications of interest (pancreatic, lung, esophageal, colorectal, head and neck, and bladder cancers), with six models included per indication. All models were treated at 10 mg/kg, which was selected as the expected maximum clinical dose of ABL209. Because high anticancer activity was observed as the experiment progressed, we added a 5-mg/kg dose group to the eight most recently initiated models to more accurately evaluate potency and dose–response relationships. A waterfall plot was generated to visualize TV changes across all evaluated PDX models (Fig. 5A; Supplementary Fig. S14). Representative EGFR and MUC1 IHC staining results of tested models are shown in Fig. 5B. ABL209 demonstrated strong antitumor activity in all the tested models. Twenty-eight models exhibited tumor regression, including 14 cases of complete regression. Additionally, the remaining eight models achieved a TGI of 70% or greater. Of the tested models with 5 mg/kg dosing, three exhibited tumor regression, whereas another three and two models achieved TGI levels of >90% and >50%, respectively. Consistent with the strong efficacy previously observed in multiple pancreatic cancer models, ABL209 also demonstrated potent activity in pancreatic cancer PDX models harboring *KRAS* mutations. Strong antitumor activity was also observed in squamous cell carcinoma of the lung, in which the TROP2-targeting ADC datopotamab deruxtecan (Dato-DXd) has reported an objective response rate of only 9.2% (25). Furthermore, tumor regression was also observed in an NSCLC model (LU5245) with a pretreatment history of docetaxel, bevacizumab, pemetrexed, and platinum-based chemotherapy.

Consistent with these efficacy results, IHC analyses revealed that EGFR and MUC1 were coexpressed in most models evaluated. This suggested that dual-targeting contributed to the strong antitumor activity of ABL209. To assess the coexpression of EGFR and MUC1 in human tumors, IHC staining was performed on a human TMA (Fig. 5C). Coexpression of the two targets was consistently observed across multiple solid tumor types, including lung, esophageal, head and neck, colon, and pancreatic cancers. These findings indicate that EGFR and MUC1 are broadly coexpressed in human malignancies. To further characterize target expression at higher resolution, scRNA-seq was performed using PDX models. Distinct cellular clusters, including discrete populations of EGFR-positive and MUC1-positive tumor cells, were observed across lung, ampullary, and pancreatic models (Supplementary Fig. S15).

### Efficacy in an osimertinib resistance PDX model

**Figure 6.**
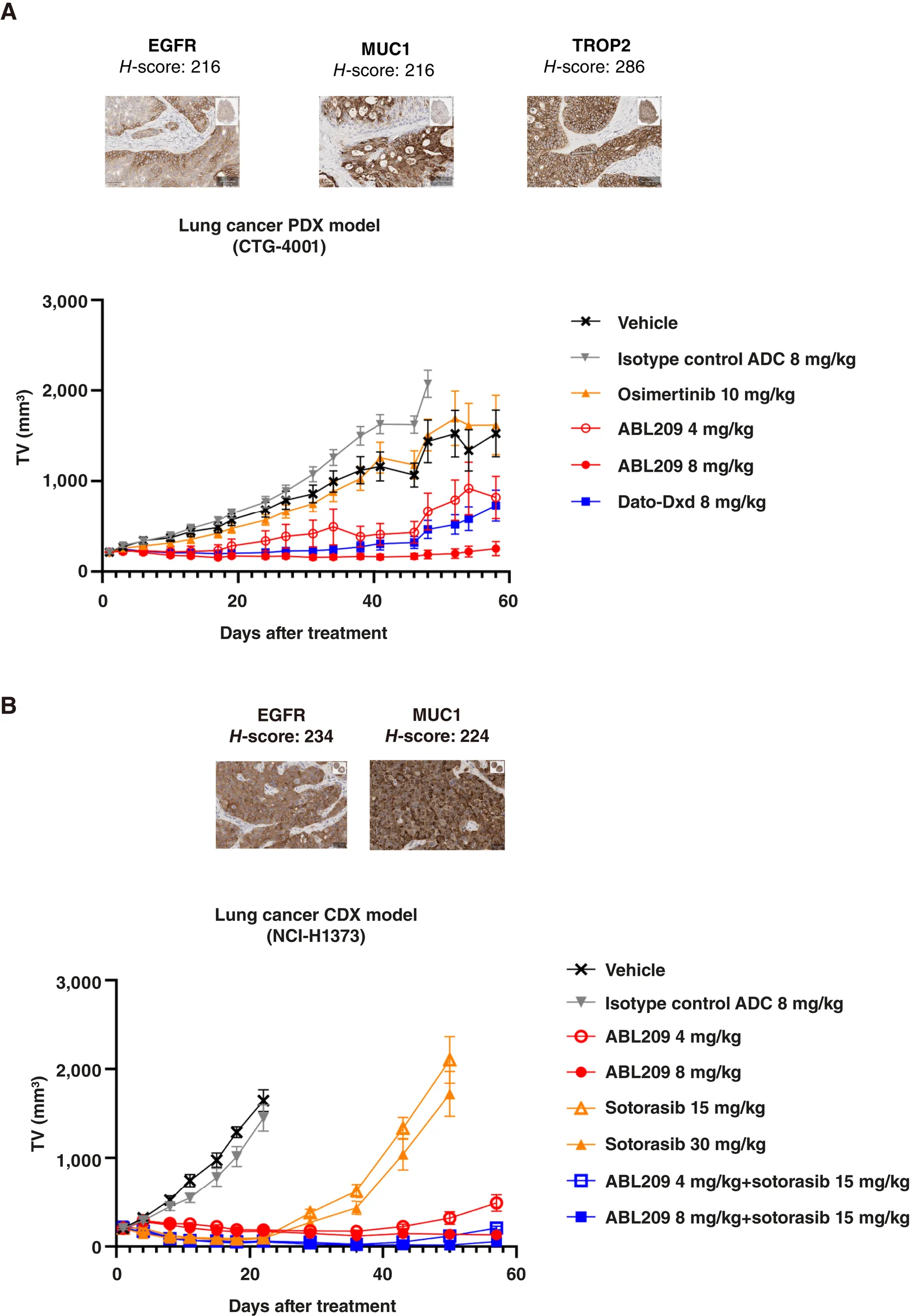
Comparative efficacy study with benchmark ADCs. **A,** Efficacy evaluation in the CTG-4001 PDX model. PDX mice were treated with ABL209 at 4 or 8 mg/kg, an isotype control ADC at 8 mg/kg, or datopotamab deruxtecan. Osimertinib was administered orally at 10 mg/kg once daily from day 0 to day 21 (*N* = 10 mice/group). Expression levels of EGFR, MUC1, and TROP2 were quantified from snap-frozen tumor samples collected at the study endpoint. **B,** Assessment of ABL209 monotherapy and sotorasib combination therapy in the NCI-H1373 NSCLC CDX model, which harbors the *KRAS* G12C mutation. ABL209 was administered intravenously at 4 or 8 mg/kg as a single dose, whereas sotorasib was administered orally once daily from day 0 to day 21 at 15 or 30 mg/kg. Combination therapy was administered according to the same dosing regimen (*N* = 7 mice/group).

We evaluated ABL209 in an NSCLC PDX model resistant to osimertinib that selectively and irreversibly inhibits the T790M EGFR mutant (Fig. 6A). On day 24 after treatment initiation, the TGI values for ABL209 and Dato-DXd at 8 mg/kg were 110% (adjusted *P* < 0.0001) and 101.3% (adjusted *P* = 0.0086), respectively. In contrast, neither osimertinib nor the nonbinding control ADC showed a significant effect on tumor growth compared with the vehicle group. As the study progressed, the difference in TV between the two ADC-treated groups became more pronounced (Supplementary Fig. S16). By day 58, only the ABL209 8-mg/kg treatment group showed a statistically significance difference from the vehicle group (adjusted *P* = 0.0039), whereas the others did not. The separation in TV continued to widen, and tumors in the ABL209 8-mg/kg group remained stably controlled. On day 58, the TGI values were 53.8% and 96.8% for ABL209 at 4 and 8 mg/kg, respectively, and 60.6% for Dato-DXd at 8 mg/kg.

### Evaluation of the potential for combination therapy with a *KRAS* inhibitor

To further establish a differentiated therapeutic strategy, we conducted an efficacy study comparing ABL209 with sotorasib, an approved *KRAS* G12C inhibitor, and evaluated the potential for combination treatment (Fig. 6B) in the NCI-H1373 CDX model, which has a *KRAS* G12C mutation. ABL209 was administered as a single intravenous dose at 4 or 8 mg/kg, whereas sotorasib was administered orally at 15 or 30 mg/kg once daily for 21 consecutive days. For the combination study, 15 mg/kg sotorasib was coadministered with ABL209 (4 or 8 mg/kg) using the same schedule as the monotherapy groups. During the dosing period, sotorasib exhibited stronger tumor growth suppression than ABL209. However, the tumor regrew quickly once sotorasib treatment was discontinued on day 22. In contrast, ABL209 showed a slower onset of antitumor activity from day 7, but sustained tumor growth was observed and did not progress until the end of the experiment (day 38). When comparing ABL209 monotherapy with the combination treatment, the combination group demonstrated quicker and sustained tumor regression. Thus, ABL209 demonstrated strong antitumor activity even in *KRAS*-mutated NSCLC models. Furthermore, the superior and sustained tumor regressions observed with combination treatment further support the clinical development of ABL209 in combination with *KRAS* inhibitors.

### Pharmacokinetics and toxicity study

To assess the pharmacokinetics (PK), ABL209 was administered once intravenously at 3, 10, and 20 mg/kg to male cynomolgus monkeys, and then the plasma concentrations of ADC, total antibody, and unconjugated exatecan were measured up to 28 days after the dose. The terminal elimination half-life (T_1/2_) of ADC at 10 mg/kg was 5.2 days. Across doses of 3, 10, and 20 mg/kg, dose-normalized AUC values were nearly identical (1.33 × 10^6^, 1.53 × 10^6^, and 1.37 × 10^6^ hours·ng/mL per mg/kg, respectively), and clearance remained consistent (0.775, 0.655, and 0.740 mL/hours/kg, respectively; Supplementary Table S4). As the PK parameters demonstrated clear dose linearity across the 3- to 20-mg/kg range, minimal target-mediated drug disposition (TMDD) can be inferred for ABL209. The PK profiles of the ADC and total antibody were nearly identical.

In a GLP toxicity study in nonhuman primates, anticipated and reversible gastrointestinal toxicity, cytopenias, and skin erythema/hyperpigmentation were observed in a dose-dependent manner. The highest nonseverely toxic dose of ABL209 in cynomolgus monkeys was established at 40 mg/kg, which is associated with a free exatecan C_max_ of 3 ng/mL.

## Discussion

The fundamental objective of an ADC is to achieve efficient, tumor-specific payload delivery. Utilizing a target that is overexpressed and essential to tumor growth offers the potential for enhanced therapeutic efficiency. EGFR is a highly relevant target that meets these criteria. However, EGFR’s broad distribution in normal tissues, notably skin and mucosa, and its involvement in normal tissue homeostasis have made the development of effective targeted therapies, such as ADCs, particularly challenging.

The 1 + 1 bispecific ADC presented in this study offers a compelling solution to this toxicity challenge. ABL209 is monovalent for each individual target, resulting in lower avidity compared with conventional bivalent antibodies. This inherently lower avidity allows the molecule to exhibit low affinity toward normal tissues that express only one target, effectively mitigating normal tissue toxicity. This is supported by the finding that ABL209 showed no detectable cytotoxicity toward primary keratinocytes and very mild skin rash (redness) in monkeys at a 20 mg/kg dose. In contrast, becotatug vedotin, a bivalent EGFR ADC, has been reported to cause skin rash in both monkeys and humans (26). In the CDX models, the bivalent MUC1 ADC showed comparable antitumor efficacy to ABL209, whereas the monovalent MUC1 ADC showed weaker or no efficacy. It can be interpreted that the bivalent MUC1 ADC has a strong cytotoxic effect against MUC1-positive cells, unlike the monovalent monospecific ADC. Such strong cytotoxicity against single-target positive cells could result in on-target toxicity against MUC1-expressing normal tissue. However, ABL209 was designed as a 1 + 1 bispecific ADC to mitigate off-tumor toxicity and related adverse effects. Notably, no toxicity was observed in the lungs and kidneys—vital organs in which MUC1 is expressed—during the monkey toxicity studies. Thus, ABL209 is expected to exhibit a more favorable safety profile than monospecific bivalent MUC1-targeting ADCs.

Although reduced valency and affinity typically compromise potency, ABL209 could overcome this limitation by leveraging high avidity through the coexpression of EGFR and MUC1. In CDX and PDX models, this bispecific ADC strategy demonstrated superior activity compared with monovalent ADCs and showed potency comparable, or even superior to, conventional monospecific bivalent ADCs. These findings confirm that the bispecific approach restores avidity and tumor-targeting specificity without sacrificing therapeutic efficacy.

TMDD and poor tumor penetration are another issue when targeting abundant antigens such as EGFR or MUC1 (27). The general approach for mitigating these issues is to increase the drug dosage. Enhertu (trastuzumab deruxtecan) is an excellent example of this strategy, successfully addressing these challenges by allowing a higher dosage of 5.4 or 6.0 mg/kg than Kadcyla (trastuzumab emtansine), in which the dose was limited to 3.6 mg/kg because of liver toxicity and thrombocytopenia, thereby surpassing the concentration threshold at which TMDD occurs (28, 29). However, the toxicity profiles of EGFR-targeting agents prevent dose escalation, indicating they remain constrained by these issues. For instance, the clinical doses for izalontamab brengitecan (EGFRxHER3 ADC; ref. 30) and becotatug vedotin are 2.5 mg/kg, and their short half-life of less than 2 days underscores these limitations. Additionally, high-density antigens can sequester high-affinity antibodies on the border ranges of the tumors, and the lower tumor penetration can limit efficacy (27). Unlike limited exposure by TMDD, this issue cannot be resolved by repeated dosing; it requires increasing the dose or reducing the affinity (31). Generally, increasing the dose tends to exacerbate toxicity, whereas reducing affinity can compromise the potency of a molecule. A 1 + 1 bispecific ADC could address this dilemma by modulating avidity for a single target and leveraging the inherent synergy of dual targeting. This architecture may allow for a higher dose because it is expected to exhibit lower avidity toward normal tissues in which only one target is present. Furthermore, the reduced affinity could be compensated for by the dual-targeting mechanism on double target–positive tumors, potentially maintaining overall efficacy. Future comparative studies with high-affinity ADCs, such as becotatug vedotin or DS-3939, are necessary to validate whether ABL209 functions through this dual-targeting mechanism.

The favorable PK and toxicity profiles of ABL209 in monkeys, together with its superior efficacy compared with the bivalent EGFR counterpart in CDX and PDX models, indicate the potential of the bispecific ADC approach to address the existing therapeutic challenges. This innovative strategy may provide a promising framework for developing ADCs against other clinically validated targets that have yet to be successfully drugged using conventional formats.

## Supplementary Material

Figure S1 Assessment of purity by SE-UPLC analysis of ADCs.

Figure S2 Assessment of DAR by RP-UPLC analysis of ADCs.

Figure S3 Amino acids sequence of ABL209.

Figure S4 Changes in body weight after the treatment in xenograft mouse models.

Figure S5 ADCC and CDC functional assays.

Figure S6 Schematic diagram of MUC1.

Figure S7 ELISA analysis using MUC1 proteins.

Figure S8 Results of cell microarray.

Figure S9 SPR sensorgrams.

Figure S10 Measurement of Caspase-3/7 activity.

Figure S11 Apoptosis analysis using Annexin V-PI staining.

Figure S12 Binding activity test of naked ABL209 and bvEGFR antibody.

Figure S13 In vivo efficacy study in the NCI-H1373 CDX model.

Figure S14 Anti-tumor activity of ABL209 in PDX models.

Figure S15 Co-expression of EGFR and MUC1 in PDX models by single-cell RNA sequencing of PDX models.

Figure S16 Tumor volume distribution in the CTG-4001 PDX model.

Table S1 Detailed information of cell lines.

Table S2 Result of SPR analysis.

Table S3 IC50 of ABL209 in various cell lines.

Table S4 PK parameters of ABL209 and total antibody in monkey plasma.

## Data Availability

The data generated in this study are available upon request to the corresponding author.
