# Supplementary material for: ABL209 (NEOK002): Designing an EGFR × MUC1 Bispecific TOP1i ADC with Promising Antitumor Activity and Enhanced Therapeutic Window
Source: Mol Cancer Ther. 2026 Jun 15;25(10):1599–613. doi: 10.1158/1535-7163.MCT-26-0088 (PMC13631502; doi:10.1158/1535-7163.MCT-26-0088)
Supplement: Supplementary file 1 [file mct-26-0088_figure_s10_supps10.docx]

**Supplementary Figure S10**

MDA-MB-468 cells was monitored in real-time using Incucyte^®^ Caspase-3/7 Green Dye after treatment of test articles. ABL209 and exatecan showed a concentration-dependent increase in caspase-3/7 activity. In contrast, treatment with non-targeting isotype control ADC or the unconjugated antibody showed no increase in green areas compared to the untreated control.
