# Supplementary material for: ABL209 (NEOK002): Designing an EGFR × MUC1 Bispecific TOP1i ADC with Promising Antitumor Activity and Enhanced Therapeutic Window
Source: Mol Cancer Ther. 2026 Jun 15;25(10):1599–613. doi: 10.1158/1535-7163.MCT-26-0088 (PMC13631502; doi:10.1158/1535-7163.MCT-26-0088)
Supplement: Supplementary file 2 [file mct-26-0088_figure_s11_supps11.docx]

**Supplementary Figure S11**

Treatment with ABL209 resulted in a significant increase in apoptotic cell populations (combined early and late apoptosis) compared with untreated controls.
