# Supplementary material for: ABL209 (NEOK002): Designing an EGFR × MUC1 Bispecific TOP1i ADC with Promising Antitumor Activity and Enhanced Therapeutic Window
Source: Mol Cancer Ther. 2026 Jun 15;25(10):1599–613. doi: 10.1158/1535-7163.MCT-26-0088 (PMC13631502; doi:10.1158/1535-7163.MCT-26-0088)
Supplement: Supplementary file 3 [file mct-26-0088_figure_s12_supps12.docx]

**Supplementary Figure S12**

Antibodies were serially diluted 4 fold from 100 nM to 0.0015 nM. Goat anti-human IgG F(ab’)2 cross-adsorbed secondary HRP antibody was used. bvEGFR is the parental bivalent monospecific EGFR antibody.
