# Supplementary material for: ABL209 (NEOK002): Designing an EGFR × MUC1 Bispecific TOP1i ADC with Promising Antitumor Activity and Enhanced Therapeutic Window
Source: Mol Cancer Ther. 2026 Jun 15;25(10):1599–613. doi: 10.1158/1535-7163.MCT-26-0088 (PMC13631502; doi:10.1158/1535-7163.MCT-26-0088)
Supplement: Supplementary file 4 [file mct-26-0088_figure_s13_supps13.docx]

**Supplementary Figure S13**

*N* = 8 mice/group were used, and all test articles were administered at 1.5 mg/kg single dose on day 1, a dose below minimum effective dose (MED) to allow accurate comparison. Each value represents the SEM. ‘mv’ means monovalent and ‘bv’ means bivalent.
