# Supplementary material for: ABL209 (NEOK002): Designing an EGFR × MUC1 Bispecific TOP1i ADC with Promising Antitumor Activity and Enhanced Therapeutic Window
Source: Mol Cancer Ther. 2026 Jun 15;25(10):1599–613. doi: 10.1158/1535-7163.MCT-26-0088 (PMC13631502; doi:10.1158/1535-7163.MCT-26-0088)
Supplement: Supplementary file 5 [file mct-26-0088_figure_s14_supps14.docx]

**Supplementary Figure S14**

Mice were treated with ABL209 intravenously at 5 mg/kg. The study was conducted at Crown Bioscience, and *N* = 3 mice/group were tested. The y-axis shows the percentage change in tumor volume relative to the initial tumor volume. Numbers on the bars are tumor growth inhibition rate.
