# Supplementary material for: ABL209 (NEOK002): Designing an EGFR × MUC1 Bispecific TOP1i ADC with Promising Antitumor Activity and Enhanced Therapeutic Window
Source: Mol Cancer Ther. 2026 Jun 15;25(10):1599–613. doi: 10.1158/1535-7163.MCT-26-0088 (PMC13631502; doi:10.1158/1535-7163.MCT-26-0088)
Supplement: Supplementary file 6 [file mct-26-0088_figure_s15_supps15.docx]

**Supplementary Figure S15**

**No**

**Yes**

A subset of tumor cells co-expressed both targets within each model (162 cells in lung, 1270 in ampullary, and 131 in pancreatic tumors), indicating the presence of double-positive tumor cell populations at single-cell resolution.
