# Supplementary material for: ABL209 (NEOK002): Designing an EGFR × MUC1 Bispecific TOP1i ADC with Promising Antitumor Activity and Enhanced Therapeutic Window
Source: Mol Cancer Ther. 2026 Jun 15;25(10):1599–613. doi: 10.1158/1535-7163.MCT-26-0088 (PMC13631502; doi:10.1158/1535-7163.MCT-26-0088)
Supplement: Supplementary file 7 [file mct-26-0088_figure_s16_supps16.docx]

**Supplementary Figure S16**

**Left**, Day 24; **Right**, Day 58. Statistical significance is indicated as follows: *p < 0.05, **p < 0.01, ***p < 0.001.
