# Supplementary material for: ABL209 (NEOK002): Designing an EGFR × MUC1 Bispecific TOP1i ADC with Promising Antitumor Activity and Enhanced Therapeutic Window
Source: Mol Cancer Ther. 2026 Jun 15;25(10):1599–613. doi: 10.1158/1535-7163.MCT-26-0088 (PMC13631502; doi:10.1158/1535-7163.MCT-26-0088)
Supplement: Supplementary file 8 [file mct-26-0088_figure_s1_supps1.docx]

**Supplementary Figure S1**

**A**

**B**

**C**

**D**

**E**

**F**

Assessment of purity by SE-UPLC analysis of ABL209 and corresponding molecules after ADC production. Panels represent the following ADCs: **A,** monovalent EGFR ADC; **B,** bivalent EGFR ADC; **C**, monovalent MUC1 ADC; **D,** bivalent MUC1 ADC; **E,** isotype control ADC; **F,** ABL209.
