# Supplementary material for: ABL209 (NEOK002): Designing an EGFR × MUC1 Bispecific TOP1i ADC with Promising Antitumor Activity and Enhanced Therapeutic Window
Source: Mol Cancer Ther. 2026 Jun 15;25(10):1599–613. doi: 10.1158/1535-7163.MCT-26-0088 (PMC13631502; doi:10.1158/1535-7163.MCT-26-0088)
Supplement: Supplementary file 11 [file mct-26-0088_figure_s4_supps4.docx]

**Supplementary Figure S4**

**A**

**B**

**C**

 **D**

Each value represents the SEM. **A,** Pancreatic CDX xenograft models. Mice were treated with ABL209 and isotype control ADC. **B,** CFPAC-1 pancreatic cancer CDX xenograft model. Mice were treated with vehicle control, ABL209, an isotype control ADC, or monospecific monovalent and bivalent ADCs. **C,** NCI-H1373 lung cancer CDX xenograft model. Mice were treated with vehicle control, ABL209, sotorasib, an isotype control ADC, or a combination of sotorasib and ABL209. **D,** CTG-4001 PDX tumors. Mice were treated with vehicle control, an isotype control ADC, ABL209, osimertinib, or datopotamab detuxtecan.
