# Supplementary material for: ABL209 (NEOK002): Designing an EGFR × MUC1 Bispecific TOP1i ADC with Promising Antitumor Activity and Enhanced Therapeutic Window
Source: Mol Cancer Ther. 2026 Jun 15;25(10):1599–613. doi: 10.1158/1535-7163.MCT-26-0088 (PMC13631502; doi:10.1158/1535-7163.MCT-26-0088)
Supplement: Supplementary file 13 [file mct-26-0088_figure_s6_supps6.docx]

# **Supplementary Figure S6**

MUC1 is cleaved into two subunits, MUC1-N (α) and MUC1-C (β), which remain associated via non-covalent interactions.
