# Supplementary material for: ABL209 (NEOK002): Designing an EGFR × MUC1 Bispecific TOP1i ADC with Promising Antitumor Activity and Enhanced Therapeutic Window
Source: Mol Cancer Ther. 2026 Jun 15;25(10):1599–613. doi: 10.1158/1535-7163.MCT-26-0088 (PMC13631502; doi:10.1158/1535-7163.MCT-26-0088)
Supplement: Supplementary file 14 [file mct-26-0088_figure_s7_supps7.docx]

**Supplementary Figure S7**

Binding activity test of ABL209 against human MUC1 proteins were evaluated by ELISA. The antigen-bound antibodies were detected with an HRP-conjugated anti-human IgG F(ab′) secondary antibody.
