# Supplementary material for: ABL209 (NEOK002): Designing an EGFR × MUC1 Bispecific TOP1i ADC with Promising Antitumor Activity and Enhanced Therapeutic Window
Source: Mol Cancer Ther. 2026 Jun 15;25(10):1599–613. doi: 10.1158/1535-7163.MCT-26-0088 (PMC13631502; doi:10.1158/1535-7163.MCT-26-0088)
Supplement: Supplementary file 15 [file mct-26-0088_figure_s8_supps8.docx]

**Supplementary Figure S8**

Binding specificity test of ABL209 and its parental antibodies using Retrogenix™ cell micro array test. Only confirmatory testing results are shown. Rituximab biosimilar was used as the positive control, and a non-binding control antibody served as the negative control for assay validation. PBS indicates secondary-only staining.
