# Supplementary material for: ABL209 (NEOK002): Designing an EGFR × MUC1 Bispecific TOP1i ADC with Promising Antitumor Activity and Enhanced Therapeutic Window
Source: Mol Cancer Ther. 2026 Jun 15;25(10):1599–613. doi: 10.1158/1535-7163.MCT-26-0088 (PMC13631502; doi:10.1158/1535-7163.MCT-26-0088)
Supplement: Supplementary file 16 [file mct-26-0088_figure_s9_supps9.docx]

**Supplementary Figure S9**

**A**

**B**

**C**

**D**

SPR analysis of ABL209 and corresponding antibodies binding to EGFR and MUC1. Sensorgrams for **A**, human EGFR; **B**, cynomolgus EGFR; **C**, human MUC1; and **D**, cynomolgus MUC1.
