# Supplementary material for: ABL209 (NEOK002): Designing an EGFR × MUC1 Bispecific TOP1i ADC with Promising Antitumor Activity and Enhanced Therapeutic Window
Source: Mol Cancer Ther. 2026 Jun 15;25(10):1599–613. doi: 10.1158/1535-7163.MCT-26-0088 (PMC13631502; doi:10.1158/1535-7163.MCT-26-0088)
Supplement: Supplementary file 17 [file mct-26-0088_table_s1_suppst1.docx]

**Supplementary Table S1**

| Cell line  (Catalog #) | Tissue | Source | RRID | Biological Sex | Culture medium |
| --- | --- | --- | --- | --- | --- |
| CFPAC-1  (CRL-1918) | Pancreatic cancer | ATCC | CVCL_1119 | Female | IMDM + 10 % FBS + 1 % penicillin/ streptomycin |
| AsPC-1  (CRL-1682) | Pancreatic cancer | ATCC | CVCL_0152 | Female | RPMI + 10 % FBS + 1 % penicillin/ streptomycin |
| HPAF-II  (CRL-1997) | Pancreatic cancer | ATCC | CVCL_0313 | Female | EMEM + 10 % FBS + 1 % penicillin/ streptomycin |
| Capan-2  (HTB-80) | Pancreatic cancer | ATCC | CVCL_0026 | Female | McCoy's 5a + 10 % FBS + 1 % penicillin/ streptomycin |
| NCI-H1373  (91373) | Lung cancer | KCLB | CVCL_1465 | Male | RPMI + 10 % FBS + 1 % penicillin/ streptomycin |
| NCI-H1975  (CRL-5908) | Lung cancer | ATCC | CVCL_1511 | Male | RPMI + 10 % FBS + 1 % penicillin/ streptomycin |
| HCC827  (CRL-2868) | Lung cancer | ATCC | CVCL_2063 | Male | RPMI + 10 % FBS + 1 % penicillin/ streptomycin |
| HT-29  (HTB-38) | Colon cancer | ATCC | CVCL_0320 | Male | McCoy's 5a + 10 % FBS + 1 % penicillin/ streptomycin |
| MDA-MB-468  (HTB-132) | Breast cancer | ATCC | CVCL_0419 | Female | L-15 + 10 % FBS + 1 % penicillin/ streptomycin |
| MDA-MB-453  (HTB-131) | Breast cancer | ATCC | CVCL_0418 | Female | L-15 + 10 % FBS + 1 % penicillin/ streptomycin |
| HCC70  (CRL-2315) | Breast cancer | ATCC | CVCL_1270 | Female | RPMI + 10 % FBS + 1 % penicillin/ streptomycin |
| CAL27  (CRL-2095) | Tongue cancer | ATCC | CVCL_1107 | Female | DMEM + 10 % FBS + 1 % penicillin/ streptomycin |
| A-431  (CRL-1555) | Skin cancer | ATCC | CVCL_0037 | Female | DMEM + 10 % FBS + 1 % penicillin/ streptomycin |
| NUGC-4  (JCRB0834) | Gastric cancer | JRBC | CVCL_3082 | Male | RPMI + 10 % FBS + 1 % penicillin/ streptomycin |
| HEKn  (PCS-200-010) | Primary human epidermal keratinocytes | ATCC | N/A | Male | Dermal Cell Basal Medium with Keratinocyte growth kit |

RRID: Research Resource Identifier, KCLB: Korean Cell Line Bank, ATCC: American Type Culture Collection

CVCL: Cellosaurus Cell Line, FBS: Fetal bovine serum, N/A: Not available
