# Supplementary material for: ABL209 (NEOK002): Designing an EGFR × MUC1 Bispecific TOP1i ADC with Promising Antitumor Activity and Enhanced Therapeutic Window
Source: Mol Cancer Ther. 2026 Jun 15;25(10):1599–613. doi: 10.1158/1535-7163.MCT-26-0088 (PMC13631502; doi:10.1158/1535-7163.MCT-26-0088)
Supplement: Supplementary file 18 [file mct-26-0088_table_s2_suppst2.docx]

**Supplementary Table S2**

| **Antigen** | **Test Article** | **Ka**  **(1/Ms, × 10^5^)** | **Kd**  **(1/s, × 10^-3^)** | **K_D_**  **(M, × 10^-9^)** | **Rmax (RU)** | **Chi^2^** |
| --- | --- | --- | --- | --- | --- | --- |
| Human  EGFR | Rituximab | No-Binding | | | | |
|  | Naked ABL209 | 3.614 ± 0.208 | 20.333 ± 0.987 | 56.277 ± 1.397 | 13.08 ± 0.81 | 0.042 ~ 0.113 |
|  | ABL209 | 3.625 ± 0.200 | 19.513 ± 2.071 | 53.730 ± 2.727 | 10.79 ± 0.71 | 0.052 ~ 0.080 |
|  | Bivalent EGFR antibody | 3.377 ± 0.039 | 21.04 ± 0.47 | 62.31 ± 2.143 | 26.47 ± 0.57 | 0.013 ~ 0.015 |
| Monkey  EGFR | Rituximab | No-Binding | | | | |
|  | Naked ABL209 | 3.723 ± 0.590 | 25.693 ± 0.592 | 69.973 ± 9.068 | 12.53 ± 1.04 | 0.013 ~ 0.091 |
|  | ABL209 | 3.816 ± 0.362 | 25.477 ± 1.221 | 66.980 ± 3.627 | 10.10 ± 0.40 | 0.055 ~ 0.080 |
| Human  MUC-1 | Rituximab | No-Binding | | | | |
|  | Naked ABL209 | 2.553 ± 0.147 | 1.064 ± 0.049 | 4.170 ± 0.186 | 19.49 ± 0.17 | 0.019 ~ 0.037 |
|  | ABL209 | 2.532 ± 0.149 | 1.345 ± 0.051 | 5.319 ± 0.126 | 18.52 ± 0.33 | 0.013 ~ 0.019 |
|  | Bivalent MUC1 antibody | 2.383 ± 0.085 | 1.017 ± 0.045 | 4.268 ± 0.035 | 39.50 ± 0.95 | 0.009 ~ 0.024 |
| Monkey  MUC-1 | Rituximab | No-Binding | | | | |
|  | Naked ABL209 | 3.565 ± 0.246 | 0.628 ± 0.016 | 1.766 ± 0.129 | 19.75 ± 0.22 | 0.032 ~ 0.077 |
|  | ABL209 | 3.560 ± 0.280 | 0.882 ± 0.010 | 2.487 ± 0.199 | 18.85 ± 0.37 | 0.013 ~ 0.049 |

Binding affinities of ABL209, bivalent EGFR antibody, bivalent MUC1 antibody, and naked ABL209 against EGFR and MUC1. Rituximab was used as the negative control, and the analysis was performed in triplicate. The human and cynomolgus MUC1 antigen used for the SPR analysis contained only the SEA domain.
