# Supplementary material for: ABL209 (NEOK002): Designing an EGFR × MUC1 Bispecific TOP1i ADC with Promising Antitumor Activity and Enhanced Therapeutic Window
Source: Mol Cancer Ther. 2026 Jun 15;25(10):1599–613. doi: 10.1158/1535-7163.MCT-26-0088 (PMC13631502; doi:10.1158/1535-7163.MCT-26-0088)
Supplement: Supplementary file 19 [file mct-26-0088_table_s3_suppst3.docx]

**Supplementary Table S3**

| **Cell line** | **IC_50_ (nM)** | | | | | |
| --- | --- | --- | --- | --- | --- | --- |
|  | **ABL209** | **bvEGFR**  **tavatecan** | **bvMUC1**  **tavatecan** | **mvEGFR**  **tavatecan** | **mvMUC1**  **tavatecan** | **Isotype control ADC** |
| MDA-MB-468 | 3.6 | 1.2 | 196.7 | 26.0 | 214.0 | 123.2 |
| HCC827 | 2.0 | 0.4 | 129.1 | 6.3 | 155.6 | 73.8 |
| HCC70 | 39.5 | N.D.* | 231.2 | 235.8 | 162.2 | 199.8 |
| NCI-H1373 | 26.8 | 78.0 | 232.6 | 117.0 | 237.6 | 143.7 |
| NCI-H1975 | 80.2 | 159.9 | 689.9 | 323.8 | > 1000 | 164.4 |
| CAL27 | 2.3 | 1.5 | 74.2 | 16.2 | 46.07 | 57.76 |
| HSC4 | 9 | 5.1 | 102 | 25.3 | 79.2 | 73.8 |
| 647V | 1.6 | 1.3 | 44.3 | 7.8 | 38.1 | 57.3 |

N.D.*: Not Determined. The 4-PL curve could not be stably fitted to the data, and the IC₅₀ value could not be accurately estimated. bv:bivalent, mv:monovalent.

Cell-based cytotoxicity assays were performed, and cell viability was used to calculate the IC_50_ value.
