# Supplementary material for: ABL209 (NEOK002): Designing an EGFR × MUC1 Bispecific TOP1i ADC with Promising Antitumor Activity and Enhanced Therapeutic Window
Source: Mol Cancer Ther. 2026 Jun 15;25(10):1599–613. doi: 10.1158/1535-7163.MCT-26-0088 (PMC13631502; doi:10.1158/1535-7163.MCT-26-0088)
Supplement: Supplementary file 20 [file mct-26-0088_table_s4_suppst4.docx]

**Supplementary Table S4**

|  | **Dose (mg/kg)** | | |
| --- | --- | --- | --- |
|  | **3** | **10** | **20** |
| **ABL209** | | | |
| **T_max_ (h)** | 0.3 (0.1 - 0.3) | 0.1 (0.1 - 0.3) | 0.1 (0.1 - 0.3) |
| **AUC_0-672h_ (h*ng/mL)** | 3990000 ± 872000 | 15300000 ± 1820000 | 27400000 ± 3840000 |
| **T_1/2_ (h)** | 96.6 (55.7 - 127.2) | 124.4 (41.3 - 137.8) | 70.1 (57.5 - 82.1) |
| **Cl (mL/h/kg)** | 0.775 ± 0.195 | 0.655 ± 0.0760 | 0.740 ± 0.109 |
| **C_max_ (ng/mL)** | 83600 ± 8950 | 322000 ± 42000 | 713000 ± 66600 |
| **V_ss_ (mL/kg)** | 82.9 ± 7.40 | 60.9 ± 6.85 | 62.1 ± 1.06 |
| **MRT_0-last_ (h)** | 110.5 (83.4 - 119.7) | 87.4 (78.1 - 101.7) | 88.8 (70.1 - 93.9) |
| **AUC/dose** | 1.33 x 10^6^ | 1.53 x 10^6^ | 1.37 x 10^6^ |
| **Total antibody** | | | |
| **T_max_ (h)** | 0.3 (0.1 - 0.3) | 0.3 (0.3 - 0.3) | 0.1 (0.1 - 0.3) |
| **AUC_0-672h_ (h*ng/mL)** | 4250000 ± 877000 | 15900000 ± 1900000 | 28400000 ± 3960000 |
| **T_1/2_ (h)** | 109.9 (59.0 - 130.2) | 140.7 (42.9 - 143.1) | 75.6 (60.4 - 89.3) |
| **Cl (mL/h/kg)** | 0.723 ± 0.171 | 0.628 ± 0.0730 | 0.711 ± 0.102 |
| **C_max_ (ng/mL)** | 84900 ± 9020 | 319000 ± 37000 | 714000 ± 49600 |
| **V_ss_ (mL/kg)** | 80.2 ± 7.00 | 62.1 ± 8.27 | 63.3 ± 1.84 |
| **MRT_0-last_ (h)** | 112.5 (88.5 - 121.8) | 93.8 (80.1 - 106.0) | 93.6 (74.6 - 97.9) |
| **AUC/dose** | 1.42 x 10^6^ | 1.59 x 10^6^ | 1.42 x 10^6^ |

ABL209 was administered once intravenously at 3, 10, and 20 mg/kg to male cynomolgus monkeys, and then plasma concentration of ADC, total antibody, and free-payload were measured up to 28 days post-dose.
