# Supplementary material for: ABL209 (NEOK002): Designing an EGFR × MUC1 Bispecific TOP1i ADC with Promising Antitumor Activity and Enhanced Therapeutic Window
Source: Mol Cancer Ther. 2026 Jun 15;25(10):1599–613. doi: 10.1158/1535-7163.MCT-26-0088 (PMC13631502; doi:10.1158/1535-7163.MCT-26-0088)
Supplement: Supplementary file 21 [file mct-26-0088_figure_s5_supps5.docx]

**Supplementary Figure S5**

**A**

**B**

Results from cell-based assays for **A**, ADCC; and **B**, CDC.
